# A Plea for a More Efficient Medical Law for Texas

**Published:** 1896-03

**Authors:** E. A. Woldert

**Affiliations:** Sec’y East Texas Medical Association, Tyler, Texas


For the Texas Medical Journal.

### A PLEA FOR A MORE EFFICIENT MEDICAL LAW FOR TEXAS.

BY E. A. WOLDERT, M. D., SEC'Y EAST TEXAS MEDICAL ASSOCIATION, TYLER, TEXAS.

Read at the meeting of the E. T. Medical Association at Tyler, Jan. 15, '96.

"'Tis hard to venture where our betters fail,
Or lend fresh interest to a twice told tale."

IT IS A generally conceded fact that the process of evolution is necessary for the progress of mankind; and we have learned by inference and experience that when a rudimentary organ of the body becomes useless, it has a tendency to retrograde.

Natural laws never change, but these laws so modify structure that it brings about such a condition that the being becomes of a higher order.

The government of human beings necessarily changes, and, from time to time, the necessary safeguards of legislation are thrown around men for the purpose of raising them to a higher standard, to restrain some and to punish others.

The existing statute in Texas, regulating the practice of medicine, was revised in 1879, seventeen years ago.

From 1880 to 1890, the population of Texas increased 71 per cent.; the value of its farms increased over 134 per cent. In 1890 she manufactured articles valued at $70,433,551, and exported goods to the value of $41,951,598, and the assessed valuation of real and personal property, in 1893, was $886,175,395.

The medical profession have also advanced in learning in all its branches. The field of knowledge developed by the microscope alone has almost caused a revolution in ancient ideas. We have, then, outgrown such a medical law, and when we say that this law is insufficient to advance the medical profession to that standard for which its forefathers consecrated their lives, we have stated the truth.

The inalienable rights of the private citizen, *guaranteed* by the Constitution of the United States, are: life (first), liberty, health, reputation, and the pursuit of happiness. Are ignorance and incompetence, on the part of a medical practitioner, a necessary guarantee of a long lease of life to the private citizen? Is it not a fact that the average quack and impostor who comes within our State is ignorant and uneducated? Because of this, is it not true that the inalienable rights of the citizen are jeopardized? Defrauding the people does not lead to improvement in the public morals. Could anything be plainer even to the average representative whom we help to elect to the legislature? "The true reason for making any act a crime is the public injury that would result." (Walker.) Do not ignorance, incompetence and false pretensions iujure the public? To say the least, the present medical law is totally inefficient, and the five thousand physicians of this State should *demand* a wiser and better law; and so should the people.

### DEFECTS OF THE PRESENT LAW.

The wording of the criminal and civil statutes is not plain; the method of appointment of the different district medical boards is not wise; the issuance of temporary and permanent certificates to undergraduates should be done away with; the words "practice of medicine" are not defined, so that prosecutions under the existing law usually fail; it does not restrain "our friends, the druggists," from prescribing promiscuously; the penalties are not severe, and the qualified physician is *therefore* harrassed by the effrontery and assurance of charlatans, empirics and quacks, who infest the public domain when the harvest is ripe. They are useless appendages to the medical economy.

### ARGUMENTS THAT HAVE BEEN PRESENTED AGAINST A MEDICAL LAW.

There is no logical reason. Because Law does not require graduation from a law school, is not a good reason that in order to practice medicine the person should not be a graduate. Law, in this country, has to do with the *acts* of individnals; medicine has to do with the *health* of the individual; is there any analogy here?

Most of the progressive States have *long ago* enacted laws which require that only graduates in medicine shall apply for license to practice; many of the older colleges require four years' attendance for graduation; most of the territories have medical laws, and the State of New Hampshire is the only State without any at all.

The howl of monopoly has often been raised when this subject has been advocated. These people confuse the words "corporation" and "monopoly." "A *corporation* is a body of persons connected together by law, either contemporaneously or in succession, and endowed with the capacity of acting for various purposes as a single man." A *monopoly*, on the other hand, "is a special privilege conferred on one or more persons to the absolute exclusion of all others." (Walker.) Corporations are not necessarily monopolies. Now, the right to practice medicine is neither a corporation nor a monopoly. It differs from a corporation from the fact that the science of medicine is *not* a creature of law. Its precepts were founded before statutes had been enacted, its precedents were established pari passu with that of common law, it became prosperous as man advanced in civilization, and would continue if no written law existed. Written law stimulates its growth by aiding evolution in bringing about a higher standard of medical education by regulating the indiscriminate practice of medicine. Its existence, then, does not necessarily depend upon written law; the medical profession will not allow it to do so, the science of medicine is too progressive for that.

Again, it is not a monopoly, because the right to practice medicine confers no special privileges on any one. Any man or woman may practice medicine without exclusion, provided they comply with the requirements of medical examinations. The field is broad, and if they are intelligent, persevering, self-sacrificing, and are willing to contribute something to that profession she is eager to receive them. Other deterring factors have recently sprung up among us; the vitapaths, physico-medacists, and Christian scientists, have somewhat retarded an otherwise healthy growth by demanding something, they know not what.

A sensible judge, in Cincinnati, recently pronounced sentence on a so-called vitapath in the following words: "Men who knowingly go into a sick room and prevent anything being done for a *dying man* by silly incantations and laying on of hands, are responsible for his death, and ought to be on a par with a *murderer* in the eyes of the law. God help the dying man who relies upon you, or any of your so-called graduates of quackery. You speak of 'vitapathy' being of a higher power than medicine, and you say you ordain ministers at the same time you matriculate vitapathic physicians. Your methods are an *insult* to intelligence, their practice is a criminal abuse of ignorance, and your colleges a disgrace to civilization."

### A FEW SUGGESTIONS RELATIVE TO THE BETTER LAW.

First of all we desire a law that will be self-supporting, for it can be carried on out of the fees provided for from applicants for license to practice. We should not ask the State for any pecuniary assistance to sustain the law; besides, the legisiature would never agree on an appropriation at this time.

If it then is self-supporting, why should it not have the support of all good citizens?

The different medical boards should be composed of the different schools of medicine, without discrimination against any school or system of medicine, each one of whom are separate and distinct from the others, and should have no affiliations, entanglements, or alliances, with politics. In this way harmony can be procured and personal interest subdued.

It is thought best by some to allow some body of men, such as the Board of Regents of the State University, to supervise the actions of the different medical boards, and to issue certificates to practice medicine. Besides providing for proficiency in medical learning, before applying for license, this board can, after a while, outline a standard which also requires the rudiments of an English education before granting licence to practice physic.

This plan has been adopted in New York, and after a trial of several years, the profession is enthusiastic over its results in weeding out those below mediocrity. The issuance of temporary and permanent certificates to applicants has been carried to such an extent that its effects are deplorable to an eminent degree, and does not reflect the credit upon the profession that it justly deserves.

It not only incapacitates, and too often stunts the growth of those to whom they are issued, but it burdens the profession in fostering their efforts without receiving but little benefit from them.

The wording should be so plain as to prevent druggists from prescribing for conditions which they do not understand, thereby endangering the public health.

The methods of enforcement of the law should be so worded that the sense will be plain, the application easy, and the penalty severe.

At the same time some provision should be made for those citizens who reside in isolated localities of the State and are deprived of the advantages which surround the more densely populated sections and where the same rigid requirements could not be employed.

### HOW THE PRESENT LAW AFFECTS THE MEDICAL DEPARTMENT OF THE STATE UNIVERSITY.

The number of matriculates at the department of medicine at Galveston was nearly two hundred at the last academic year; the number of applicants for graduation was not many and the number of graduates were about half a dozen, because many do not see fit to complete the course when they can practice medicine on a certificate. This deprives the State University of that revenue which she so richly deserves; and, therefore, proper provision should be made for its protection, if for nothing else. The principal sources of maintenance of this institution is a very small portion of the interest on bonds to the value of $575,840, and on land notes of the University, and a few thousand dollars from tuition fees,* with such appropriations as the legislature can be induced to make. The University of Texas must live to educate representatives. Had the University private donations, such as some of the older universities, the University of Pennsylvania, for instance, which received $1,115,000 within a month, she might be more fully equipped.

*There are no "tuition fees."—Ed.

With a deficit in the treasury, how can the State make any appropriations for this seat of learning?

A deficit in the treasury usually means a tax on somebody, which falls as well upon the physician as it does upon the average citizen, the exception in the case being these impostors who feast upon the hard earned savings of the simple minded.

Texas, like all other States without good medical laws, has been, is now, and will become the resort of that class of parasites who apply invectives to the qualified physician and who sap the essence of vitality from the fraternity without giving value received, and transport to other fields that remuneration which should go to make her own citizens more proficient in their work.

Why will representatives be so negligent of the real interests of the people as to be swayed by this class of men who are not their friends or supporters?

### HOW CAN WE OBTAIN RELIEF?

Whole volumes have been written on this subject alone. The best efforts have been futile. Men, grown old in the work, have lost courage, and say "that the public schools of the State have not been in existence long enough."

The representatives render only faint praise to those who are most earnest in this work; do not see the necessity for it; do not care for it anyhow, and often make light of the zeal displayed by its supporters.

The main trouble, it seems, has been that the medical profession has lacked organization and united support of any bill, while the opponents have presented a solid phalanx.

The profession of the State are devided in their opinions as to what the law should be. "In union there is strength," but the opposite now seems to reign as the opprobrium medicorum.

If any one doubts this, let him try it and see. The representatives think that if the doctors can not agree on what they want, themselves, they should not have anything. If we expect anything, organization should take place at once, in order to find out what is wanted. Let the State Medical Association sanction it; petition the governor to mention it in his message to the next legislature, and insist that it be passed with few or no amendments. The desires of the homeopaths and electics should also be determined before the bill is sent to the legislature.

What the representatives want to know is, if it is a popular measure? Does the petition have the sanction of the majority of the people? For that is what they are sent to Austin for; not to labor for a few, but for the *common welfare* of its citizens.

Another question seems to be, is the medical profession strong and powerful enough to demand the support of each representative before their election? Several, who have had more experience in this matter than the speaker, claim that we can, and if such is the case, we, the physicians representing the different schools of medicine of this State should agree and define definitely what we want, and *demand* of each representative, before pledging our vote, that they will support a proper medical bill.

We have learned who our enemies are, and should know how to fight them, and, having the support of the representatives, the conquest should not be prolonged.

### WHAT SHALL THE CAPTION BE?

Some physicians have argued that we could not have a bill passed whose caption reads "An Act to Regulate the Practice of Medicine." They state that the representatives say "the physicians have no right to regulate anything." It is granted without any argument whatever that the physicians can not regulate the practice of medicine. No good argument can be adduced to prove that they should. The *law* regulates the indiscriminate practice of medicine, while the physicians aid in enforcing it, and this custom should so continue.

The physicians do not want to create a monopoly.

The caption only states what follows in the body of the law; and following in the wake of twenty-four States in this Union we should state openly and with no covert terms what we are fighting for, and let the people be the arbiters in this matter.

The following has been compiled as being of interest in this connection:

States which provide that graduades (only) may apply for examination: Arizona, California, Florida, Idaho, Minnesota, Nebraska, Nevada, New York, Ohio, Delaware, Pennsylvania, South Carolina.

States and Territories which permit any person to present themselves for examination: Arkansas, Colorado, Illinois, Indiana, Iowa, Kentucky, Maine, Mississippi, Missouri, New Mexico, New Jersey, Alabama, North Carolina, Oregon, Tennessee, Virginia, West Virginia.

States and Territories which permit the State Board of Health to issue or approve licenses: Illinois, Iowa, Kentucky, Louisiana, Mississippi, Missouri, Nebraska, New Mexico, West Virginia.

States and Territories, in which the governor appoints the Board of Medical Examiners who issue licenses: West Virginia —Two members from each senatorial district, and who serve four years; Utah—Seven members; New Mexico—Seven members, who serve two years each; Colorado—Nine members, who serve two, four and six years, respectively; Florida—Board for each district to serve four years; Minnesota—Nine members, who serve one, two and three years, respectively; New Jersey—Nine members, who serve one, two and three years, respectively; Oregon—Three members, who serve one, two and three years, respectively; Tennessee—Six members; Utah—Seven members; Virginia—Two members from each senatorial district, and who serve four years.

State in which the different medical societies nominate the medical examiners, who are subsequently appointed by the governor: Pennsylvania—Twenty-one members, and who serve one, two and three years, respectively.

State in which the different medical societies nominate the medical examiners, and who are appointed by the State Board of Regents: New York—Twenty-one members, and who serve three years.

State in which the regular physicians are the sole examiners for license: North Carolina—Seven members, who are chosen by the State Medical (Regular) Society.

States in which the medical examiners are appointed by the different societies and who grant licenses: Colorado—Seven members, who serve one year; Maryland—Fourteen members, who serve four years.

States and Territories which permit females to practice midwifery without examination: Arkansas, Indiana, Maryland, Mississippi, New Mexico; Tennessee, Virginia, West Virignia, Texas.

State which allows the circuit court to grant licenses: Indiana.

States which have written examinations only: Iowa, New Jersey, New York, Pennsylvania.

State which has no medical laws: New Hampshire.

States which have taxed itinerent vendors of medicine: California, Connecticut, Illinois, Iowa, Missouri, Nebraska, Oregon, Tennessee, West Virginia.

Fees for licences in the different States and Territories: West Virginia, $10; Arkansas, $6; California, $5; Colorado, $5; Florida, $10; Illinois, $5; Iowa, $10; Maryland, $10; Minnesota, $10; Mississippi, $10; New Jersey, $15; New Mexico, $20; New York, $10; North Carolina, $10; Pennsylvania, $25; Tennessee, $10; Utah, $25; Virginia, $5; Texas, $15.

States and Territories, whose caption reads "An Act to Regulate the Practice of Medicine;" surgery also included in same: Arizona, Arkansas, California, Colorado, Florida, Georgia, Illinois, Iowa, Louisiana, Minnesota, Missouri, Montana, Nebraska, Nevada, New Jersey, New Mexico, North Dakota, South Dakota, Oregon, Tennessee, Texas, Utah, Virginia, Washington.

---

[The foregoing statistics, relating to the medical laws of States, are taken from Polk's Directory for 1891.]

